# Determination of Morphological, Biometric and Biochemical Susceptibilities in Healthy Eurasier Dogs with Suspected Inherited Glaucoma

**DOI:** 10.1371/journal.pone.0111873

**Published:** 2014-11-07

**Authors:** Thomas Boillot, Serge G. Rosolen, Thomas Dulaurent, Frédéric Goulle, Philippe Thomas, Pierre-François Isard, Thierry Azoulay, Stéphanie Lafarge-Beurlet, Mike Woods, Sylvie Lavillegrand, Ivana Ivkovic, Nathalie Neveux, José-Alain Sahel, Serge Picaud, Nicolas Froger

**Affiliations:** 1 Clinique Vétérinaire Vetea, Saintes, France; 2 INSERM, U968, Institut de la Vision, Paris, France; 3 Sorbonne Universités, UPMC Univ Paris 06, UMR_S 968, Institut de la Vision, Paris, France; 4 CNRS, UMR 7210, Institut de la Vision, Paris, France; 5 Clinique Veterinaire Voltaire, Asnières sur Seine, France; 6 Centre Hospitalier Veterinaire Saint-Martin, Saint-Martin de Bellevue, France; 7 Clinique Veterinaire Aquivet, Eysines, France; 8 Clinique Veterinaire Saint-Pierre, Saint-Chamond, France; 9 Clinique Veterinaire des Halles, Strasbourg, France; 10 Vébio, Arcueil, France; 11 Pimrose Hill Veterinary Clinic, Dun Laoghaire, Co. Dublin, Ireland; 12 Department of Nutrition, Faculty of Pharmacy, Paris Descartes University, Paris, France; 13 Clinical Chemistry, Hôtel-Dieu-Cochin Hospitals, AP-HP, Paris, France; 14 Fondation Ophtalmologique Adolphe de Rothschild, Paris, France; 15 Centre hospitalier d′ophtalmologie des Quize-Vingts, Paris, France; 16 University College London, London, United Kingdom; Université de Lorraine, France

## Abstract

In both humans and dogs, the primary risk factor for glaucoma is high intraocular pressure (IOP), which may be caused by iridocorneal angle (ICA) abnormalities. Oxidative stress has also been implicated in retinal ganglion cell damage associated with glaucoma. A suspected inherited form of glaucoma was recently identified in Eurasier dogs (EDs), a breed for which pedigrees are readily available. Because of difficulties in assessing ICA morphology in dogs with advanced glaucoma, we selected a cohort of apparently healthy dogsfor the investigation of ICA morphological status, IOP and plasma concentrations of oxidative stress biomarkers. We aimed to establish correlations between these factors, to identify predictive markers of glaucoma in this dog breed. A cohort of 28 subjects, volunteered for inclusion by their owners, was selected by veterinary surgeons. These dogs were assigned to four groups: young males, young females (1–3 years old), adult males and adult females (4–8 years old). Ocular examination included ophthalmoscopy, tonometry, gonioscopy, biometry and ultrasound biomicroscopy (UBM), and the evaluation of oxidative stress biomarkers consisting of measurements of plasma glutathione peroxidase (GP) activity and taurine and metabolic precursor (methionine and cysteine) concentrations in plasma. The prevalence of pectinate ligament abnormalities was significantly higher in adult EDs than in young dogs. Moreover, in adult females, high IOP was significantly correlated with a short axial globe length, and a particularly large distance between Schwalbe's line and the anterior lens capsule. GP activity levels were significantly lower in EDs than in a randomized control group of dogs, and plasma taurine concentrations were higher. Hence, ICA abnormalities were associated with weaker antioxidant defenses in EDs, potentially counteracted by higher plasma taurine concentrations. This study suggests that EDs may constitute an appropriate canine model for the development of glaucoma. This cohort will be used as a sentinel for longitudinal monitoring.

## Introduction

Glaucoma, the second most common cause of blindness worldwide [1], is a group of ophthalmic diseases characterized by the loss of retinal ganglion cells (RGCs) whose function is to transfer visual information to the brain through the optic nerve. High intraocular pressure (IOP) is considered a major risk factor for glaucomatous neuropathies [2]. IOP level is partly regulated by the rate of aqueous humor (AH) formation, which normally equals the rate of outflow. Abnormalities in AH turnover have been implicated in most types of hypertensive glaucoma [3]. However, a progressive loss of vision can occur in patients with normal tension as well as when their IOP is controlled with drugs [4], [5], and oxidative stress is now well known to play a critical role in RGC degeneration [6]. This role has been suggested by (i) the decrease in systemic glutathione levels in patients with primary open-angle glaucoma [7] and (ii) the association of this form of glaucoma with a polymorphism of the glutathione S-transferase gene [8]. The importance of combating oxidative stress to ensure RGC survival was confirmed by the prevention of RGC degeneration by taurine, a major antioxidant [9].

The dog, an animal of intermediate size with an ocular anatomy and physiology similar to that of humans and living in the same environment as humans, may constitute a suitable model for the investigation of susceptibilities to glaucoma because spontaneous glaucomas are observed in certain dog breeds [10], [11], [12], [13], [14]. As in humans, the primary risk factor for glaucoma in dogs seems to be high IOP [15], also regulated by AH turnover [15], [16], [17]. AH drainage mechanisms involve the iridocorneal angle (ICA) and the anterior opening of the ciliary cleft (CC), which are spanned by the comb-like pectinate ligament. The passage of AH between the intraligamentary spaces leads to its entry into the uveal and then the corneoscleral trabecular meshwork before being collected by the angular aqueous plexus, the intrascleral plexus, and the vortex venous drainage system [15]. Drainage via this conventional route accounts for 85% of AH outflow in dogs [18]. Pectinate ligament abnormalities (PLA), involving a lack of resorption of the mesenchymatous tissue filling the anterior part of the ciliary cleft during embryonic development, have often been reported in association with primary narrow and closed-angle glaucoma [10], [19]. Primary glaucomas are further classified as having an open or narrow ICA on the basis of either gonioscopic examination [20] or imaging by ultrasound biomicroscopy (UBM) [21], [22].

The underlying genetic susceptibilities potentially contributing to the development and progression of primary glaucoma have never been clearly identified. Glaucoma is genetically heterogeneous, and many genes have been reported to be linked to primary open-angle glaucoma in both humans and dogs [23], [24], [25], [26]. In several dog breeds, including the Chow Chow [27], the Samoyed [28], and the Keeshound [29], primary glaucoma is considered to be hereditary with a prevalence of 4.7% and 1.6%, in Chow Chow and Samoyed dogs, respectively [30]. These two breeds were used to generate Eurasier dogs (EDs): a breed obtained in the early 1960s by Julius Whipfel in Germany and characterized by a small population size and intense inbreeding.

In a preliminary study, we observed high rates of blindness in EDs from France, associated with the clinical signs of glaucoma (e.g. mydriasis, episcleral congestion and diffuse corneal edema) and high IOP [31]. We therefore established a cohort of apparently healthy EDs to determine whether these dogs displayed any morphological and biochemical features conferring a predisposition to glaucoma. Our ultimate objective was to determine whether the ED could be considered a genuine animal model of glaucoma for subsequent therapeutic and genetic investigations.

## Materials and Methods

Dogs were included in the study by veterinary surgeons from the European *Réseau Européen d′Ophtalmologie Vétérinaire et de Vision Animale* (REOVVA) network.

The specific protocols were approved by the joint ethics committee of the Vision Institute, the Pierre et Marie Curie University and REOVVA. All animals were examined with the consent of their owners.

### Animal cohorts

Three cohorts of dogs were recruited by the REOVVA network:

1. The first cohort (cohort 1) was used in the preliminary study investigating the blindness associated with high IOP that occurs in EDs.
2. Imaging was not possible in the animals of the first cohort due to severe corneal edema and mydriasis. We therefore established a second cohort (cohort 2) of apparently healthy EDs.
3. The third cohort (cohort 3) of randomized healthy dogs of various breeds was established for a comparison of biochemical parameters with those of the second cohort.

#### Glaucomatous dogs (cohort 1)

Cohort 1 was identified by clinical examination. All animals were presented by their owners for an abnormal appearance of the eyes and vision problems. Ophthalmic examinations were performed and included indirect ophthalmoscopy, slit-lamp examination and IOP measurements. This cohort included 18 dogs (11 males and 7 females from 14 generations; age: 6.7± 1.0 years old; mean±SEM).

##### *Apparently healthy EDs (cohort 2)*

We ensured that this cohort included a representative distribution of EDs by including dogs from 8 generations of a family of 830 individuals. The family tree was constructed with GenoPro software according to data obtained for pedigrees from the website “Chiens de France” (cross-linked sources). Dogs were selected from various lineages and were volunteered for participation by their owners. The dogs were obtained from different breeders and owners from across the country, but the rate of interbreeding was nevertheless as high as 60% in some individuals. The selected dogs were healthy and had no apparent macroscopic clinical signs of glaucoma (e.g., mydriasis, episcleral congestion, diffuse corneal edema). The normal range of IOP has not been determined for this breed, so we did not use IOP as an inclusion criterion. Cohort 2 included 28 dogs (age: 4.0±0.6 years old; means±SEM) divided into homogeneous groups on the basis of age and sex: 7 young males (age: 1–3 years old; weight: 20–30 kg), 9 young females (age: 1–3 years old; weight: 16–26 kg), 6 adult males (age: 4–8 years old; weight: 24–31 kg), and 6 adult females (age: 4–8 years old; weight: 20–26 kg).

##### *Control dogs (cohort 3)*

An additional cohort of healthy dogs was used as a control for biochemical comparisons with EDs. This cohort was randomized for sex, age, weight and breed. It included 39 dogs (15 males; 24 females; age: 3.10±1.7 years old; weight: 20.5±2.2 kg; means±SEM) from 25 different breeds other than the ED such that the values could not be biased by a breed with specific features. The breeds included in this control cohort were not known to be prone to either primary ocular or any other diseases. For example, we did not include the Basset Hound, the American Cocker Spaniel or the Welsh Terrier, three breeds known to develop primary glaucoma, in this cohort. Blood samples were collected primarily for reasons unrelated to this study.

### Clinical examinations and tonometry (cohorts 1, 2)

Complete ocular examinations were performed on all animals, including slit-lamp examination, indirect ophthalmoscopy (with 20D and 30D lenses), and tonometry. We harmonized procedures and decreased individual variation by systematically performing ophthalmoscopy with devices equipped with small-pupil systems to prevent pupil dilation [32]. This was considered important because it is not possible to evaluate the pectinate ligament precisely once the pupil has become dilated. The optic nerve head was evaluated by indirect ophthalmoscopy with a Volk Macula Plus 5.5X for enlargement. IOP was always determined at the same time of day, between noon and 3 p.m., to minimize the effects of circadian variations [33], [34], with a rebound tonometer (Tono-Vet, Tiolat, Espoo, Finland). The IOP values recorded for each eye were the mean of three successive tonometer readings on the central cornea.

### Gonioscopy (cohort 2)

Before bilateral gonioscopy, the cornea was anesthetized by the topical application of proxymetacaine (Proparacaine 0.5%, Bausch & Lomb, Chauvin Pharmaceuticals Ltd., Aubenas, France). Direct gonioscopy was performed with a 17 mm Koeppe goniolens (Ocular Instruments, Bellevue, WA, USA) filled with hydroxyethylcellulose fluid (Gelaser, Alcon, Rueil Malmaison, France) before being placed in contact with the cornea, ensuring that there were no air bubbles or nictitating membrane entrapment. Examinations were performed on conscious animals whenever possible, but anesthesia was used if necessary (induction: 2 µg/kg medethomidine, 5 mg/kg ketamine intramuscularly, followed by the inhalation of 1.5 to 3% isoflurane gas in a mixture of 30% air and 70% oxygen). General anesthesia likely influences the ICA morphology, but there is no reference in dog because gonioscopy is a qualitative examination.

The anesthetized animals were placed in a lateral recumbent position with the head positioned so as to ensure that the plane of the iris was parallel to the table. The ICA was viewed with a hand-held slit-lamp biomicroscope (Kowa SL-2, SL-14 or SL-15). The entire ICA and the opening of the CC were systematically examined for the presence of PLAs. Goniophotography was performed according to a standardized procedure (DSLR camera +100 mm macro lens + Koeppe lens 17 mm + Gelaser + Finoff transilluminator) for each eye and each quadrant. In cases of PLA detection, the abnormality was quantified by assigning a percentage of the 360° after systematically viewing the entire circumference of the ICA, and a grade was attributed according to the ICA percentage. All quantifications were carried out by the same investigator (Thomas Dulaurent) who received the data without any additional information about age, sex, breed or animal status. The grading used corresponds to that recommended by the French Eye Panellists/Maladies Héréditaires Oculaires Canines (AFEP/MHOC)] Association eye scheme.

### Ultrasonography (cohort 2)

B-mode ultrasonography (10 MHz probe, OTI, EDC Vet, Carvin, France) was performed on each animal and on each eye to measure the axial globe length (AGL) from the corneal epithelial surface to the inner retinal surface. Only one image per eye was selected by veterinarians and used for measurements. Images were sent to the same investigator (Thomas Dulaurent) to minimize the individual coefficient of variation in the analysis.

### Ultrasound biomicroscopy (UBM) (cohort 2)

For each animal, and for each eye, a 48 MHz (Accutome, UBM-Plus, Malvern PA, USA) monotransducer with geometric focalization and line scanning was used for this study. As previously described, anesthetized animals were positioned in a recumbent position with the head stabilized with a hollow cushion [22]. A series of video sequences was recorded and sent to the same investigator (Thomas Dulaurent) in each case, and he selected one image among 20 images, which optimally demonstrated the maximal distance between the corneal endothelium in the region of the vertex and the most anterior portion of the length capsule. For analyses, the transducer was positioned in the central region of the anterior chamber to measure the anterior chamber length (ACL), determined as the distance between the corneal endothelium in the region of the corneal vertex and the anterior pole of the lens. For ICA parameter measurements, outflow pathways were evaluated in the dorsal quadrant. The transducer was placed perpendicularly to the corneoscleral limbus, in the 12 o′clock position, and scanning was then carried out from the 10 to 2 o′clock positions.

### Collection of blood samples (cohorts 2, 3)

For determinations of biomarkers of inflammation and oxidative stress, we collected blood samples (5 ml) from the cephalic veins of fasted, awake animals. Half the blood sample was used for the extraction of plasma which was then immediately frozen at -20°C.

### Determination of biomarkers of inflammation and oxidative stress (cohorts 2 & 3)

Inflammatory biomarkers, including albumin, C-reactive (CRP) and haptoglobin, were determined in the plasma by spectrophotometry with diagnostic kits (previously validated in dogs) and controls from Thermofisher, and a KONELAB 60 (Thermofisher) clinical chemistry analyzer. These biomarkers were selected as representative of the different phases of the inflammatory response [35], [36]. CRP is a biomarker of the very early phase (several hours) whereas haptoglobin increases at a slower rate (few days) but remains high for longer amounts of time. By contrast, albumin synthesis decreases during inflammation.

Glutathione peroxidase (GP) activity in the plasma was assessed by spectrophotometry with a diagnostic kit and controls from Randox Laboratories and a KONELAB 60 (Thermofisher) clinical chemistry analyzer. Finally, plasma concentrations of taurine, methionine and cysteine were determined by ion exchange chromatography, as previously described [37].

### Statistical analysis (cohort 2, 3)

For each ED subgroup, qualitative data are expressed as the proportion (%) of examined eyes considered to be abnormal. These proportions were compared in chi-squared (χ^2^) tests to identify significant differences in the distribution of these events between subgroups. Quantitative data are presented as means ± SD. We used the non-parametric Mann & Witnney test to compare mean values between two groups. For simultaneous comparisons of more than two groups, we carried out the non-parametric Kruskal-Wallis comparison of variance, followed, if significant, by a Dunn's post-hoc test to compare the means of the groups considered. Differences were considered significant if *p*<0.05.

We used the non-parametric Spearman's rank correlation test to detect non-linear correlations between parameters. The correlation was considered significant if *p*≤0.05. Linear correlation analyses were also carried out and were considered significant if r^2^>0.52.

## Results

### Clinical examination and tonometry (Cohort 1, 2)

#### Cohort 1

Diagnosed by an initial exam, EDs from cohort 1 displayed clinical signs of glaucoma including diffuse corneal edema, episcleral congestion, and mydriasis. These dogs included ten (4 males and 6 females) with an IOP>35 mmHg who were already blind, and eight (7 males and 1 females) with an IOP of between 25 and 35 mmHg who were not blind. When compared with the IOP value in normal dogs (19.0 mmHg with a range of 11 (5%) and 29 (95%) mmHg [38]), this cohort had elevated levels and many individuals exceeded the limit>35 mmHg defined as abnormal by Gelatt et al. [15]. Due to the severe corneal edema and mydriasis, neither gonioscopy nor UBM could be performed in these dogs to assess ICA morphology.

#### Cohort 2

Because we undertook the first study in healthy EDs it was important to mention all ocular abnormalities and IOP levels. On clinical examination, none of the animals examined presented clinical signs of glaucoma (e.g. mydriasis, episcleral congestion, diffuse corneal edema), but 25.0% of the eyes were found to display ocular abnormalities other than those specifically related to glaucoma and identifiable with the AFEP/MHOC scheme (see below).

The prevalence of these abnormalities was higher (i) in females than in males and (ii) in adults than in young dogs (**Table 1**). The prevalence of clinical eye abnormalities was significantly higher in adult females (33.3%) than in young females (*p*<0.05; χ^2^ test; **Table 1**).

**Table 1:** Ocular abnormalities and tonometry in the cohort of healthy Eurasier dogs (cohort 2).

Clinical Observations and tonometry	Male dogs (*N* = 13)	Female dogs (*N* = 15)	Young dogs (*N* = 16)	Adult dogs (*N* = 12)	Young males (*N* = 7)	Young females (*N* = 9)	Adult males (*N* = 6)	Adult females (*N* = 6)
Eyelid abnormalities	2	3	5	-	2	3	-	-
Corneal dystrophies	-	2	-	2	-	-	-	2
Lens opacities	3	1	1	3	-	1	3	-
Micropapilla	1	-	1	-	1	-	-	-
Choroidal hypoplasia	-	2	-	2	-	-	-	2
Total abnormalities (%)	6 (23.1%) (n = 26)	8 (26.7%) (n = 30)	7 (21.9%) (n = 32)	7 (29.2%) (n = 24)	3 (21.4%) (n = 14)	4 (22.3%) (n = 18)	3 (25.0%) (n = 12)	4 (33.3%) (n = 12)
IOP (mm Hg)	17.7±4.5 (*n* = 26)	15.7±3.4 (*n* = 29)	17.8±4.2 (*n* = 31)	15.5±3.5 (*n* = 24)	18.8±5.2 (*n* = 14)	16.9±2.9 (*n* = 17)	16.5±3.1 (*n* = 12)	14.4±3.6 (*n* = 12)

Abnormalities were observed on clinical examinations other than gonioscopy examination. Data represent the numbers for each category of abnormalities observed in each subgroup of EDs and the total numbers of abnormalities (with a percentage) are presented in the last line.

Tonometric data are expressed as means ± standard deviation (SD).

*n* refers to the number of eyes examined in each group whereas *N* is the number of dogs in each group.

IOP: intraocular pressure.

Mean IOP values for each group were towards the upper limit of the normal range for dogs with a similar morphology [39], [40], [41]. No significant differences in IOP levels were found between the ED subgroups (p = 0.16, Kruskall-Wallis ANOVA, **Table 1**). However, a surprising decrease of IOP (−13%) was found in adult EDs when compared to young EDs (**Table 1**). An even larger decrease was observed in the analyses restricted to females (−15%; **Table 1**). Regarding the glaucoma prevalence in EDs (see cohort 1 above), healthy dogs with IOP of 25–35 mmHg are not considered clinically glaucomatous because normal IOP ranges are unknown in EDs.

### Gonioscopy (Cohort 2)

**Figure 1:**
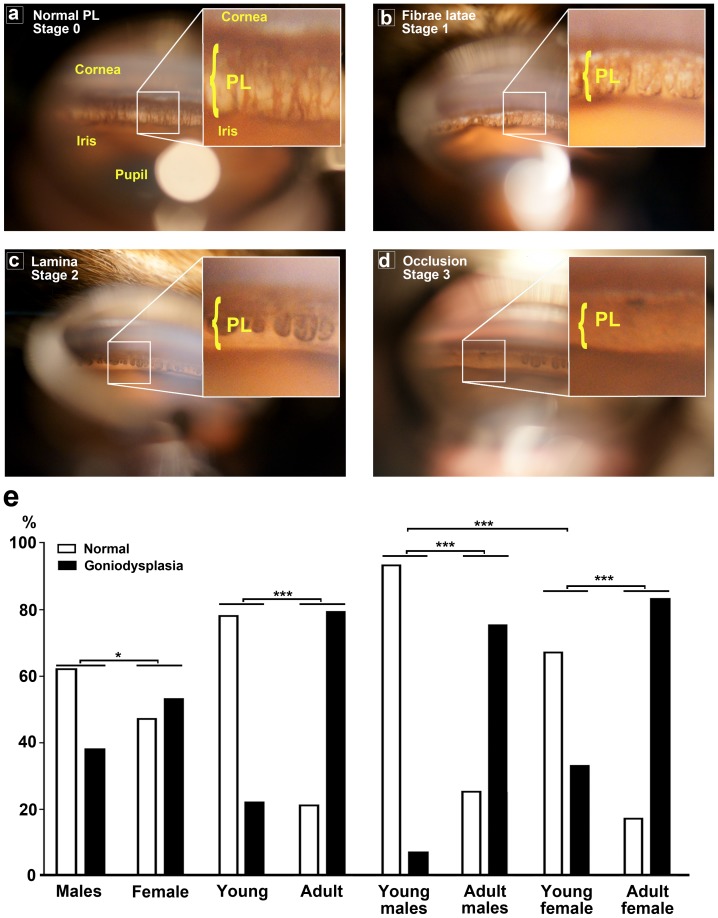
Pectinate ligament abnormalities (PLA) evaluated by gonioscopic examination in Eurasier dogs. (a) Normal pectinate ligament (PL) visible when rarefaction of the initial fibrilar sheet was almost complete, by 2 to 4 weeks after birth, leaving strands of intertwining collagen, progressively encased by attenuate trabecular cells, confluent with the anterior surface of the iris (stage 0). (b-d) The entire ICA and opening of the CC were systematically examined for the presence of a PLA, defined as abnormally broad and thickened pectinate ligament fibers (stage 1) (**b**) or solid sheets (stage 2) (**c**) of PL tissue, with or without “holes” (stage 3) (**d**). (e) Percentage of PLA (including grade 1, 2 and 3; black bars) with respect to normal pectinate ligament (white bars) in each category of the ED cohort. *p<0.05 and ***p<0.001 indicate significant differences in the prevalence of PLA between the indicated groups (χ^2^ test).

When the ICA was viewed with a hand-held slit-lamp biomicroscope (**see** **Fig. 1a**), the entire ICA and opening of the ciliary cleft were systematically scrutinized for the presence of PLA **(** **Fig.1b-d** **)**. Such abnormalities were rated as being at different stages of progression: stage 1 (fibrae latae) corresponded to abnormally broad and thickened pectinate ligament fibers; stage 2 (lamina) corresponded to the appearance of solid sheets of pectinate ligament tissue; stage 3 (occlusion) was characterized by the persistence of an embryonic sheet of ICA tissue and an absence of intraligamentary spaces.

For PLA quantification, the grades were as follow: grade 0 corresponded to an absence of PLA **(** **Fig. 1a** **)**; grade 1 corresponded to a mild (stage 1 and/or stage 2; **Fig. 1b**) PLA affecting less than 25% of the ICA; grade 2 corresponded to a moderate PLA (stage 1 and/or stage 2) involving between 25% and 75% of the ICA circumference (stage 2; **Fig. 1c** **)**; grade 3 corresponded to severe PLA (stage 1 and/or stage 2 affecting more than 75% of the ICA and/or the presence of an occlusion), regardless of the percentage of the circumference affected (stage 3; **Fig. 1d** **)**. PLA scoring was performed by an independent investigator on anonymous pictures to ensure the attribution of an unbiased grade.

The prevalence of PLA was higher in females (53.3%) than in males (38.4%) in an analysis of all grades together (*p*<0.05, χ^2^ test; **Fig. 1e**). However, males were more frequently affected by the minor forms of PLA (grade 1: 60.0%), whereas females displayed more severe forms of PLA (grades 2 & 3: 56.3%; **Table 2**).

**Table 2:** Morphological appearance of the pectinate ligament in the cohort of healthy Eurasier dogs (cohort 2).

PL aspects (%)	Male dogs (*N* = 13)	Female dogs (*N* = 15)	Young dogs (*N* = 16)	Adult dogs (*N* = 12)	Young males (*N* = 7)	Young females (*N* = 9)	Adult males (*N* = 6)	Adult females (*N* = 6)
**Grade 0 (normal)**	16	14	25	5	13	12	3	2
**Grade 1**	6	7	2	11	1	1	5	6
**Grade 2**	1	3	1	3	0	1	1	2
**Grade 3**	3	6	4	5	0	4	3	2

The PL was observed by gonioscopy. In cases of pectinate ligament abnormalities, quantification was performed by assigning a percentage of the 360° after systematically viewing the entire circumference of the ICA, and a grade was attributed according to the percentage of the ICA affected (AFEP/MHOC). Grade 0 corresponds to an absence of goniodysplasia (see figure 1a), grade 1 corresponds to mild goniodysplasia (stage 1 in figure 1b), grade 2 corresponds to moderate goniodysplasia (stage 2 in figure 1c), and grade 3 corresponds to severe goniodysplasia (stage 3 in figure 1d). PLA grades are provided separately for the two eyes because PLA did not progress uniformly in the two eyes.

Data are expressed as the number of eyes affected by the various grades of goniodysplasia for each group of EDs. *N* is the number of dogs in each group.

Interestingly, the frequency of PLA increased with age; as such abnormalities were found in 79.2% of adult EDs and only in 21.8% in young EDs (*p*<0.001, χ^2^ test; **Fig. 1e**). Minor forms of PLA (grade 1) were more prominent in adult EDs (45.8%), whereas the more severe forms (grades 2 & 3) were observed mostly in young EDs (15.7%; **Table 2**). In addition, PLA was more prevalent in young females (33.4%) than in young males (7.2%; *p*<0.001, χ^2^ test; **Fig. 1e**), and young females displayed predominantly severe forms (27.8% for grades 2 & 3). By contrast, in adult EDs, PLA affected both sexes equally (75% for males and 83.4% for females; **Fig. 1e**), but the forms detected were essentially minor (50% grade 1 for females and 41.7% for males; **Table 2**).

### Biometry and UBM examinations (Cohort 2)

**Figure 2:**
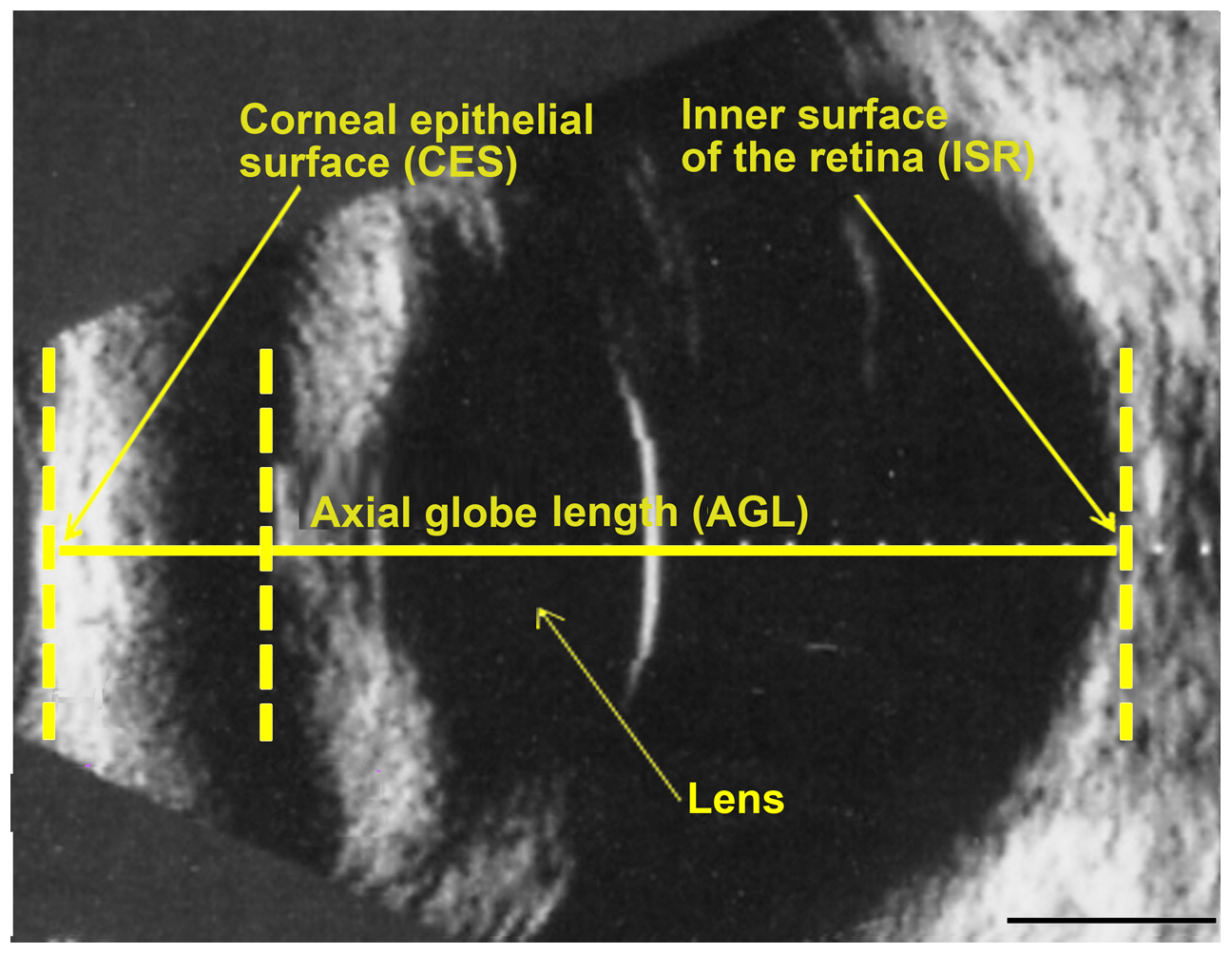
Biometry of the dog eyeball. Representative image from a normal dog obtained by B-mode ultrasound: a standard 10 MHz transducer was placed directly on the cornea after the instillation of topical anesthetic into the eye. A vertical axial scan was performed, making it possible to measure axial globe length (AGL) — the length from the corneal epithelial surface (CES) to the inner surface of the retina (ISR). The normal lens is translucent and only the posterior lens capsule is visible, as a highly reflective concave line. The anterior chamber and the vitreous are clear and anechoic and appear as homogeneous black areas. The scale bar represents 1 mm.

Biometric examinations were carried out to measure (i) AGL, defined as the distance between the corneal epithelial surface and the inner retinal surface (**figure 2** **)**, and (ii) ACL, defined as the distance between the corneal endothelium at the corneal vertex and the anterior pole of the lens. Analyses of biometric results revealed significant differences in AGL between the different ED subgroups considered (*p*<0.01; Kruskall-Wallis ANOVA; **table 3**). In particular, AGL was found to be significantly lower in female EDs than in male EDs (*p*<0.05, Dunn's post-hoc test; **table 3**). This decrease in females was found to be more significant when compared to adult males (p<0.001, Dunn's post-hoc test; **table 3**). In males, AGL of adults was significantly higher (*p*<0.01, Dunn's post-hoc test) than that of young dogs **(** **Table 3** **)**. By contrast, no significant difference in ACL was found between any of the subgroups considered (**Table 3**).

**Table 3:** Biometry and UBM measurements for the cohort of healthy Eurasier dogs (cohort 2).

Biometric measurements	Male dogs (*N* = 13)	Female dogs (*N* = 15)	Young dogs (*N* = 16)	Adult dogs (*N* = 15)	Young males (*N* = 7)	Young females (*N* = 9)	Adult males (*N* = 6)	Adult females (*N* = 6)
AGL (mm)	19.9±0.6 (*n* = 26)	19.5±0.6* (*n* = 26)	19.6±0.5 (*n* = 28)	19.9±0.7 (*n* = 24)	19.6±0.6 (*n* = 14)	19.5±0.5 (*n* = 14)	20.3±0.5°°° ^¶¶^ (*n* = 12)	19.5±0.8^##^ (*n* = 12)
ACL (mm)	3.7±0.2 (*n* = 26)	3.8±0.4 (*n* = 26)	3.8±0.2 (*n* = 28)	3.8±0.4 (*n* = 24)	3.7±0.3 (*n* = 14)	3.8±0.3 (*n* = 14)	3.7±0.2 (*n* = 12)	3.9±0.5 (*n* = 12)
CCL (mm)	2.6±0.6 (n = 25)	2.5±0.6 (*n* = 28)	2.5±0.5 (*n* = 31)	2.6±0.7 (*n* = 22)	2.5±0.5 (*n* = 14)	2.4±0.5 (*n* = 17)	2.6±0.8 (*n* = 11)	2.6±0.7 (*n* = 11)
CCW (mm)	0.27±0.09 (*n* = 25)	0.25±0.09 (*n* = 28)	0.24±0.09 (*n* = 31)	0.28±0.08 (*n* = 22)	0.23±0.10 (*n* = 14)	0.25±0.09 (*n* = 17)	0.30±0.06 (*n* = 11)	0.26±0.09 (*n* = 11)
DSAC (mm)	2.8±0.2 (*n* = 20)	2.8±0.3 (*n* = 27)	2.9±0.3 (*n* = 26)	2.8±0.2 (*n* = 21)	2.9±0.2 (*n* = 10)	2.9±0.3 (*n* = 16)	2.8±0.2 (*n* = 10)	2.8±0.2 (*n* = 11)

Data are expressed as means ± standard deviation (SD) and *n* is the number of eyes examined in each group whereas *N* is the number of dogs in each group. We did not include all the eyes, because some of the images could not be exploited.

AGL: axial globe length; ACL: anterior chamber length; CCW: ciliary cleft width; CCL: ciliary cleft length; DSAC: distance from Schwalbe's line to the anterior capsule of the lens.

^*^ *p*<0.05 versus the male group; °°°p<0.001 versus females; **^##^** *p*<0.01 versus the adult males and **^¶¶^** *p*<0.01 versus the young male group (Kruskal-Wallis ANOVA followed by a Dunn's post-hoc test).

**Figure 3:**
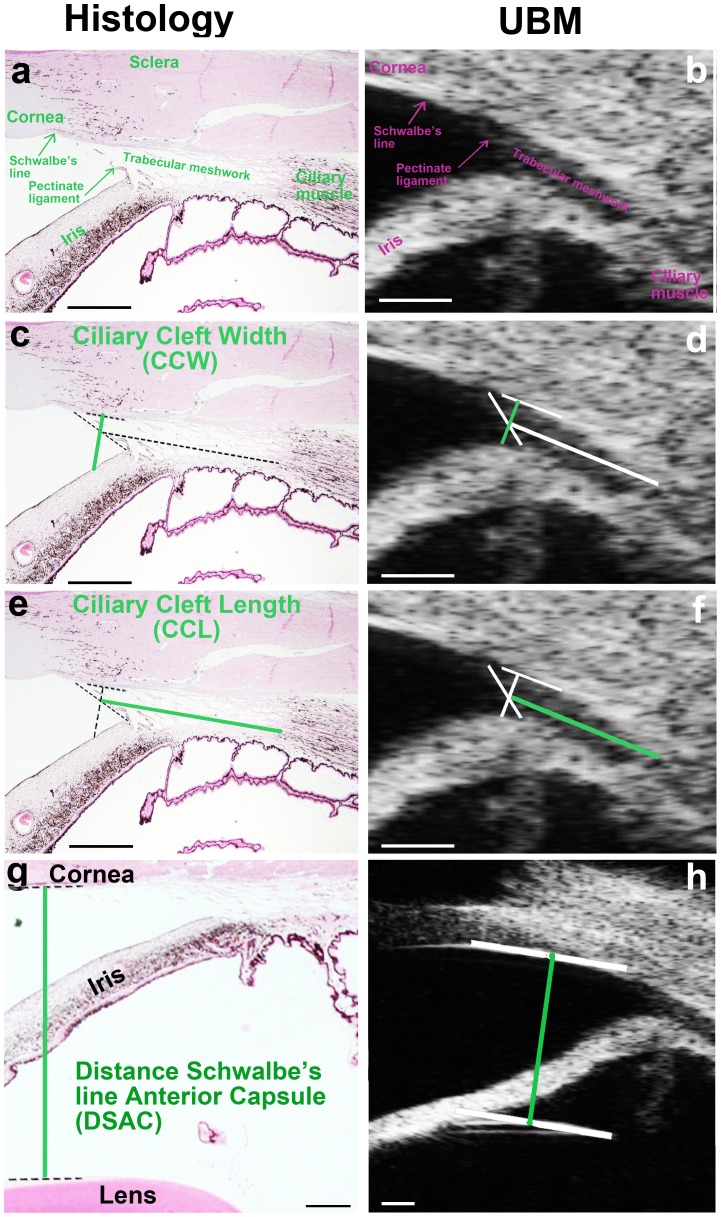
Iridocorneal angle (ICA) in dogs: images from ICA sections versus ultrasound biomicroscopy. (a-b) Normal histological sections of the ICA (stained with hematoxilin/eosin) are shown on the left, with the corresponding UBM images on the right. ICA structures in (a) are compared with those obtained by UBM (b). The pectinate ligament corresponds to the anterior delineation of the ciliary cleft. The trabecular meshwork, which fills the ciliary cleft (CC), is clearly visible. (c-d) The width of the entrance of the ciliary cleft (CCW) was measured by determining the distance between the corneoscleral limbus and the iris root as follows. A line outlining the pectinate ligament was traced. Another line, outlining the inner wall of the sclera in the anterior portion of the CC, was then traced. The CCW corresponds to a line perpendicular to the inner wall of the sclera and crossing the pectinate ligament halfway along its length. (e-f) The length of the CC (CCL) was determined as the distance from the pectinate ligament (or the most anterior visible portion of the uveal trabecular meshwork) to the anterior portion of the ciliary body as follows. A line was traced outlining the pectinate ligament. The CCL corresponds to a line parallel to the long axis of the CC, crossing the pectinate ligament halfway along its length at the anterior and reaching the ciliary muscle at the posterior. (g-h) Determination of the distance between Schwalbe's line (the peripheral termination of Descemet's membrane, i.e. the borderline between the cornea and sclera) and the anterior capsule of the lens (DSAC). The scale bar corresponds to 1 mm for all images. The ICA sections were obtained from the same dog as the UBM images.

The “en face” CC opening can only be visualized by gonioscopy, whereas the entire cleft can be imaged by ultrasound biomicroscopy (UBM) **(** **Fig. 3 a-b** **)**, allowing the measurement of its width and length. Three parameters were evaluated for each eye, as described in **figure 3**: (i) the width of the entrance of the CC (CCW), determined as the distance between the corneoscleral limbus and the iris root **(** **Fig. 3c,d** **)**, (ii) the length of the CC (CCL), determined as the distance between the pectinate ligament (or the most anterior visible portion of the uveal trabecular meshwork) and the anterior portion of the ciliary body **(** **Fig. 3e-f** **)** and, finally, (iii) the distance between Schwalbe's line (the peripheral termination of Descemet's membrane, i.e., the borderline between the cornea and sclera) and the anterior capsule of the lens (DSAC) (**Fig. 3g-h**). This last distance is considered a suitable parameter for the comparison of UBM measurements between dogs with different body sizes/weights [42].

No significant difference in the mean values of CCW, CCL and DSAC was found between the subgroups of EDs **(** **Table 3** **)**. Thus, UBM measurements indicated that the anatomy of the CC was unaffected by age or sex in the cohort of EDs studied (cohort 2).

### Correlation between IOP biometry and UBM parameters (Cohort 2)

We investigated the possible effect of changes in biometric and UBM parameters on changes in IOP; and vice versa, by carrying out statistical tests to evaluate the correlation between IOP and quantitative parameters assessing ICA morphology (biometric and UBM parameters, respectively).

**Figure 4:**
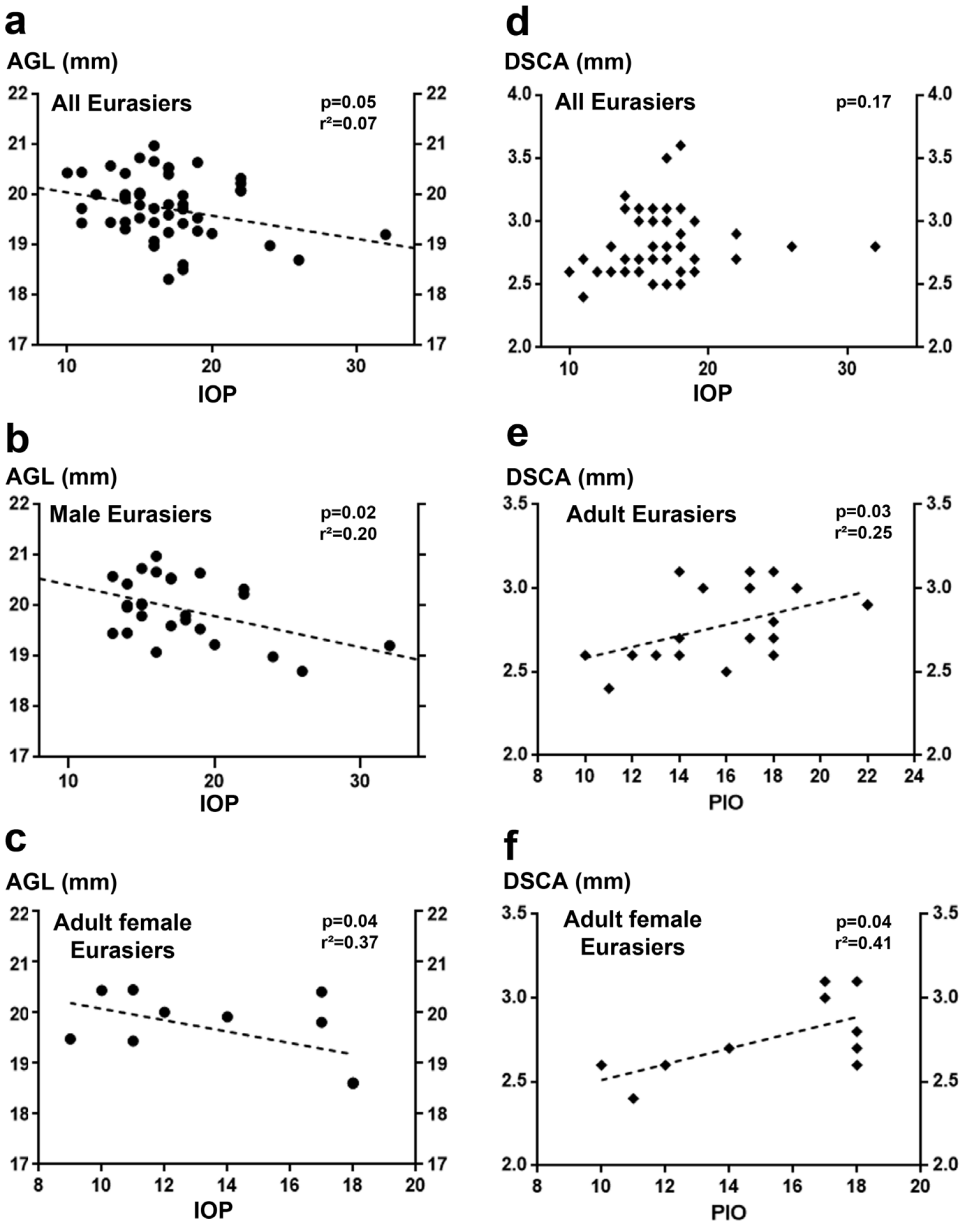
Correlation between intraocular pressure (IOP) and axial globe length biometric parameters in the various subgroups of the ED cohort. (a-c) Correlation between IOP and axial globe length (AGL) in all EDs from the cohort: increasing IOP was significantly associated with decreasing AGL (*p* = 0.05, Spearman's test; a). Similar significant correlations were also found for adult EDs (*p* = 0.03; b) and for female adult EDs (*p* = 0.05; c). The linear regression analysis (hatched lines) demonstrated the non-linear status of the correlations, because *r* ^2^<0.5 in all cases (a-c). (d-f) Correlation between IOP and distance between Schwalbe's line and the anterior capsule of the lens (DSAC): increasing IOP was significantly associated with increasing DSAC in male EDs (*p* = 0.03; e) and adult female EDs (*p* = 0.05; f), whereas no significant correlation between these parameters was found when all EDs were considered together (*p* = 0.17; d). Linear regression analysis (hatched lines) indicated that the significant correlations observed were not linear (*r* ^2^<0.52 in all cases; d-f).

A plot of the IOP levels of all EDs against biometric parameters (AGL, ACL) revealed a significant inverse correlation between IOP and AGL (**Fig. 4a**), suggesting that a decrease in AGL may lead to an increase in IOP (*p* = 0.05; Spearman's test). Similar correlations were found for analyses limited to the male ED subgroup (**Fig. 4b**) and the adult female ED subgroup (**Fig 4c**).

In an analysis that included all EDs, no significant correlation was found between IOP and the UBM parameter: DSAC (*p* = 0.17; Spearman's test; **Fig 4d**). However, we found a significant positive correlation between these parameters for the adult subgroup (**Fig. 4e**) and for adult females (**Fig. 4f**). This observation indicates that decreases in IOP may be associated with decreases in DSAC, or vice versa.

### Biochemical investigations (Cohort 2, 3)

Concentrations of various biomarkers of inflammation (albumin, CRP, haptoglobin) and oxidative stress (GP activity, taurine and its precursors: methionine and cysteine) were measured in the plasma of healthy EDs (cohort 2) and compared to those measured in the control dogs (cohort 3).

No significant differences in concentrations of inflammatory biomarkers were found between these two cohorts of dogs. Similarly, no differences in the levels of these biomarkers were found between the various ED subgroups (**Table 4**). These results suggest that levels of inflammatory biomarkers in EDs are identical to those in the general dog population.

**Table 4:** Biochemical markers of inflammation determined in plasma from the cohort of healthy Eurasier dogs (cohort 2).

Biochemical markers	Male dogs (*N* = 13)	Female dogs (*N* = 15)	Young dogs (*N* = 16)	Adult dogs (*N* = 12)	Young males (*N* = 7)	Young females (*N* = 9)	Adult males (*N* = 6)	Adult females (*N* = 6)
Albumin (g/l)	30.0±2.0	29.6±2.3	29.5±2.4	30.2±1.7	30.3±2.3	28.8±2.4	29.7±1.7	30.7±1.8
CRP (mg/l)	6.3±1.7	6.8±1.6	6.5±1.7	6.7±1.6	5.9±1.1	7.0±2.1	6.8±2.3	6.5±0.5
Haptoglobin (g/l)	0.7±0.4	0.2±0.2	0.4±0.4	0.6±0.4	0.6±0.4	0.2±0.2	0.8±0.3	0.3±0.3

Data are expressed as means ± standard deviation (SD) for each group. *N* is the number of dogs in each group.

CRP: C-reactive protein.

**Figure 5:**
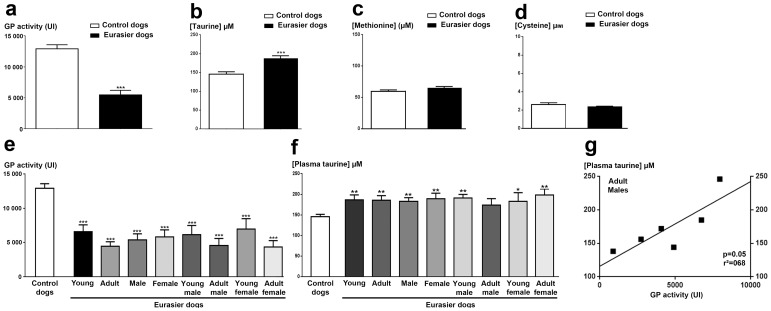
Determinations of biomarkers of oxidative stress in the plasma of Eurasier dogs. (a) Plasma glutathione peroxidase (GP) activity in all EDs (black bar), compared with a cohort of randomized control dogs (white bar). (b) Measurement of plasma taurine concentrations in EDs (black bar), compared with control dogs (white bar). (c-d) Determination of the concentrations of the taurine's precursors, methionine and cysteine, in plasma from EDs (black bar) and control dogs (white bar). (e): Measurement of plasma GP activity in the various subgroups of the ED cohort. (f): Measurement of plasma taurine concentration in the various subgroups of EDs. (g) Correlation between GP activity and plasma taurine concentration in the adult male subgroup of EDs, demonstrating a significant association of low levels of GP activity with a low plasma taurine concentration (*p*<0.05; Spearman's correlation test). This correlation was shown to be linear (r^2^>0.52). ***p<0.001 versus the control dogs (Mann Whitney test for a, b,c and Kruskal-Wallis ANOVA followed by the Dunn's post-hoc test for d, e, f).

GP activity was significantly lower in healthy EDs than in the control dogs (**Fig. 5a**). This enzymatic activity was also significantly lower in each of the subgroups of EDs than in the control dogs (*p*<0.001, Kruskal-Wallis ANOVA followed by a Dunn's post-hoc test; see **Fig. 5e**). GP activity was stronger in young than in adults EDs, but this difference was not statistically significant (**Fig. 5e**).

Surprisingly, plasma taurine concentrations were higher in EDs than in the control dogs (*p*<0.001, Mann & Whitney test; **Fig. 5b**). By contrast, the plasma concentrations of methionine and cysteine were similar in the EDs and the control dogs (**Fig. 5c-d**). Each subgroup of EDs displayed a significantly higher plasma taurine concentration than the control dogs, except for the adult male subgroup (**Fig. 5f**). This suggests that such increase in plasma taurine concentration was not dependent on age or sex in healthy EDs, but instead is typical of the breed. However, the lower plasma taurine concentration observed in the adult male EDs was linearly correlated (*p* = 0.05 and *r* ^2^ = 0.68) with the lower level of GP activity, suggesting an increased vulnerability to oxidative stress with age in male EDs.

## Discussion

Primary glaucoma has been reported to occur at a high prevalence in various dog breeds which is consistent with the existence of hereditary forms [27] [28] [29] [30]. We recently published the first report of high rates of spontaneous glaucoma affecting both sexes of adult EDs in France [31], as shown by severe visual problems and clinical signs of glaucoma on clinical examination, together with high IOP.

In a cohort of healthy EDs without a diagnosis of glaucoma, we found that IOP values were towards the upper end of the mean values obtained for dogs with Tonovet [39], [40], [43], but that these values tend to decrease with age. Because no reference describing IOP ranges in EDs is available, we have no explanation for the decline of IOP with age, although a similar phenomenon was already described in other breeds [38]. Clinical investigation revealed the presence of ocular abnormalities in all ED subgroups, regardless of age or sex. PLA accounted for 50% of all abnormalities and was more frequent (∼80%) in adult EDs, suggesting that changes in ICA morphology may be correlated with aging in this dog breed, particularly in females. This observation was already described in another breed (Flat coated retriever), but remains under debate, and it cannot exclude an effect of the breed selection [44]. The relationship between glaucoma and PLA has never been clearly established in dogs, although it was suspected by some studies in various breeds [11], [20], [44], [45]. In addition, « dysplasia » is a controversial notion amongst veterinary ophthalmologists because theoretically a normal dysplasia cannot change with time. Surprisingly, young dogs displayed more severe forms of PLA than adult dogs, suggesting the possibility of changes in the appearance of the pectinate ligament over time in dogs, consistent with a recent study in another dog breed (Flat-Coated Retriever) [46]. The high prevalence of minor forms of PLA (grade 1) in adults in our study may be accounted for by the progression of asymptomatic forms (grade 0) to minor forms (grade 1), but this hypothesis requires confirmation in a longitudinal follow-up study of this cohort. The higher prevalence of PLA in female EDs suggests that females are more prone to the development of primary glaucoma [15], as previously reported in other breeds [47] and even in humans, with women being more frequently affected than men [1]. This finding is also consistent with the reported two-fold higher risk of primary angle closure glaucoma in females than in males [48], [49].

Very few studies including biometric UBM analyses for ICA phenotyping have been performed in dogs, and no such studies have been performed on EDs. Our data therefore provide the reference values for this breed. We found that AGL (i) increased with age, particularly in males, and (ii) was higher in males than in females, probably due to the higher size/weight ratio in males (27.3±3.7 kg in males versus 21.8±2.7 kg in females; *p*<0.001), as described in Samoyeds [28]. Regarding the higher value of AGL in adult males, similar result was described in normal eyes Samoyed [50], without any rationale to explain such a phenomenon. The other parameters studied (ACL, CCL and CCW) displayed no significant change with aging. We found an inverse correlation between IOP and AGL, particularly for the adult female subgroup (high IOP and low AGL). This suggests that the smaller AGL in female EDs may render them more susceptible to the development of a high IOP. A more surprising positive correlation was found between DSAC and IOP in adult females. With age, the lens increases in volume [43], [51], [52] and may move backwards, towards the posterior chamber of eye. The anterior capsule of the lens is thicker and more rigid in dogs [53] than in humans [54], potentially resulting in equatorial and posterior lens deformation, keeping the ACL constant but decreasing the DSAC. High IOP has been identified as a major risk factor for the initiation and development of glaucoma, but the correlation of IOP with biometric parameters (AGL and DSCA) suggests that changes in these parameters could serve as alternative biomarkers and/or risk factors for the development of glaucoma. Furthermore, these correlations were more marked in adult female EDs, a subgroup particularly affected by PLA. Accordingly, these dogs present ICA abnormalities which are relevant to the development of glaucoma.

Oxidative stress has been identified as a major mechanism underlying the development of glaucoma and RGC degeneration [6]. The importance of this role was highlighted by the decrease in systemic glutathione concentrations observed in patients with primary open glaucoma [7] and the association of this form of glaucoma with a polymorphism of the glutathione S-transferase M1 gene [8]. The activity of GP, a crucial enzyme for the glutathione cycle [55], was very low in EDs. Taurine, another important retinal antioxidant [56], has also recently been reported to play a major role in RGC survival [9]. Surprisingly, we found that plasma taurine levels were higher in EDs than in the control dogs, whereas methionine and cysteine concentrations were similar in these two groups. Taurine is endogenously involved in the process of ROS detoxification in mitochondria [57]. The plasma taurine concentrations may be higher due to an increase in taurine transporter activity responsible for the plasma taurine concentrations. Given the powerful antioxidant action of taurine, these high plasma taurine levels may compensate for the lower level of GP activity. However, the linear correlation between GP activity and plasma taurine concentration suggests that this compensatory effect of taurine may change with age, which possibly would render EDs more susceptible to oxidative stress as adults.

EDs may therefore constitute a genuine canine model for glaucoma. Unlike human patients, the ED breed of dogs is characterized by a very high percentage of inbreeding, which should facilitate the search for the genetic origins of the ICA and glutathione phenotypes. However, it is necessary to study a larger number of dogs to assess the genetic transmission of these phenotypes. The cohort studied here is now being monitored longitudinally with the genotyping of putative candidate genes. In addition, therapeutic treatments, such as beta-blockers and/or carbonic anhydrase inhibitors and/or prostaglandins, will be proposed to animals showing an apparition of glaucomatous symptoms.

We aim to investigate the correlation between ICA modifications and glaucoma development and to identify the cause of susceptibility to glaucoma. This work is based on the notion that there are biomarkers of glaucoma common to humans and dogs, consistent with involvement of the same causal genes in the two species [58]. This study thus provides an illustration of the “one disease, one medicine” concept [59], to the mutual benefit of humans and dogs [60].
